# The Microscopic Anatomy of the Human Body, in Health and in Disease

**Published:** 1855-01

**Authors:** 


ART. VII.— *The Microscopic Anatomy of the Human Body, in Health and in Disease. Illustrated with numerous drawings in color.* By Arthur Hill Hassall, M. B., Author of "History of the British Fresh Water Algæ," F. L. S., M. R. C. S., etc., etc. With additions to the Text and Plates, and an Introduction containing Instructions in Microscopic Manipulation. By Henry Van Arsdale, M. D. In two volumes. New York: Samuel S. & William Wood. 1855.

The multiplication of practical works on General Anatomy is a feature of medical process which must afford pleasure to every lover of medical science. It is another of those constantly recurring indications that the days of hypothesis and of doubtful or erroneous physiology are drawing to a close; that hereafter we are to believe what is proved, and leave the uncertain open for investigation.

Those unfamiliar with microscopic research can have but little idea of the difficulties which environ it. Place a microscope in the hands of a tyro and he knows not how to "work it;" teach him this, and his eye must still be educated to the ocular illusions which are developed by high magnifying powers and the refraction of light.

But after all, the microscope is only a highly magnified eye. Long, tedious, and delicate manipulations are necessary before a tissue can be so isolated or arranged as to make the microscope available. Its focus directed upon an unprepared organ reveals nothing of its structure.

Preparatory to subjection to the microscope, its vessels must be injected, a process so delicate that even in good ha ıds the failures exceed the successes, and then follows the dissection, the thin slice so diaphanous that light shall readily pass through it, or the opaque object with its depth and richness of color and outline, the "mounting" in cell, or in balsam, or dry, the getting rid of air bubbles, and the final hermetical closure of the little cavity—all these are but a tithe of the difficulties which obstruct the study of general anatomy.

All these the microscopist, by patience and long practice, learns to conquer, and finally to present, as the reward of weary toil, the single mounted specimen which reveals the intimate structure so painfully sought and obtained.

We have spent most of the morning in reading the introduction to the treatise under notice. So far as our limited familiarity with the practical difficulties of the art enables us to judge, its directions for injections, dissections, and mountings, are extremely useful. Of the text of Hassall's Anatomy we cannot speak with precision. So far as we have examined, (here and there opening to a passage,) it is marked by simplicity of description, and by scientific accuracy in argument. The additions by Dr. Van Arsdale, seem also to be valuable and important.

Thus far we have spoken of the first volume in such terms as our acquaintance with it justifies. The second volume, (like Gerber's General Anatomy and Quekett's Histology,) is an atlas of the first, illustrating by plates and figures the tissues described.

It is not too much to say that with these we are delighted. Engravings of microscopic objects are usually dry subjects of study, but these are different. The coloring is magnificent—not too high, nothing extravagant about it, but truthful, neat, accurate, and faithfully representing the objects as we have sometimes seen them in the choicest specimens of Parisian mounting. To say that they do away with the microscope itself would be too much, but they are certainly the best substitutes which have ever come under our notice.

We could instance a series of colored drawings of the minute anatomy of the liver as being peculiarly elegant, or of the kidneys, equally elegant, but not taken from quite so perfect injections.

We have seen, for instance, a triple injection of a kidney, in which the arteries and malpighian tufts were red, the veins blue, and the tubuli urineri white, thus displaying the whole structure of the gland at a single view beneath the microscope. Had the artist given us such a drawing in the beautiful coloring which his other sketches possess, the illustration of the kidney would have been as complete as that of the liver.

A large number of these drawings, (and some of the finest of them,) are additions to the American edition. Among them is a series of the changes taking place in Bright's disease of the kidneys, which are extremely valuable.

In conclusion of this—the most decided and unqualified puff we have committed in a long time—we can only ask our readers to remember that we do not always or indiscriminately praise works sent us by publishers.

The Messrs. Woods deserve much credit for the beautiful style in which they have gotten up this edition, and we hope that a large and profitable sale may reward them. H.

